# Older adult experience of care and staffing on hospital and community wards: a cross-sectional study

**DOI:** 10.1186/s12913-020-05433-w

**Published:** 2020-06-26

**Authors:** Kirsten Barnicot, Kirsty Allen, Chloe Hood, Mike Crawford

**Affiliations:** 1 Mental Health Services Research, City University of London, School of Health Sciences, Myddleton Building, 1 Myddleton Street, London, EC1R 1UW UK; 2 Division of Psychiatry, Imperial College London, London, UK; 3 West London NHS Trust, London, UK; 4 Royal College of Psychiatrists, London, UK

**Keywords:** Healthcare quality improvement, Hospital medicine, Nurses, Patient-centred care, Patient satisfaction

## Abstract

**Background:**

Recent major concerns about the quality of healthcare delivered to older adults have been linked to inadequate staffing and a lack of patient-centred care. Patient experience is a key component of quality care - yet there has been little research on whether and how staffing levels and staffing types affect satisfaction amongst older adult hospital inpatients. This study aimed to evaluate the association between registered nurse and healthcare assistant staffing levels and satisfaction with care amongst older adult hospital inpatients, and to test whether any positive effect of higher staffing levels is mediated by staff feeling they have more time to care for patients.

**Methods:**

Survey data from 4928 inpatients aged 65 years and older and 2237 medical and nursing staff from 123 acute and community medical wards in England, United Kingdom (UK) was collected through the Royal College of Psychiatrist’s Elder Care Quality Mark. The cross-sectional association between staffing ratios and older adult patient satisfaction, and mediation by staff perceived time to care, was evaluated using multi-level modelling, adjusted for ward type and with a random effect for ward identity.

**Results:**

Higher numbers of patients per healthcare assistant were associated with poorer patient satisfaction (adjusted β = − 0.32, 95% CI − 0.55 to 0.10, *p* < 0.01), and this was found to be partially mediated by all ward staff reporting less time to care for patients (adjusted β = − 0.10, bias-corrected 95% CI − 1.16 to − 0.02). By contrast, in both unadjusted and adjusted models, the number of patients per registered nurse was not associated with patient satisfaction.

**Conclusions:**

Older adult hospital patients may particularly value the type of care provided by healthcare assistants, such as basic personal care and supportive communication. Additionally, higher availability of healthcare assistants may contribute to all ward staff feeling more able to spend time with patients. However, high availability of registered nurses has been shown in other research to be vital for ensuring quality and safety of patient care. Future research should seek to identify the ideal balance of registered nurses and healthcare assistants for optimising a range of outcomes amongst older adult patients.

## Background

A number of high profile reports have highlighted deficiencies in hospital care for older adults, and this has been linked to low numbers of nursing staff and a lack of patient-centred care [1–4]. The Royal College of Nursing has responded with recommendations for minimum staffing levels on wards, specifying a maximum number of patients per staff member of any type and per registered nurse, and a maximum number of healthcare assistants (who are unregistered and for whom no formal training is required) per registered nurse [5]. However, when the UK National Institute for Health and Care Excellence looked for evidence to back up safe staffing guidance on acute adult inpatient wards, they judged it to be of poor quality [6].

Patient experience is increasingly recognised as the third pillar of healthcare, alongside clinical effectiveness and safety [7], and its assessment is considered mandatory for driving quality improvement [8]. Patient satisfaction is inherently important to patients and their families, and central to clinicians’ aim of providing humanistic patient-centred healthcare. Satisfaction is also an indicator of quality of care, predicting both clinical safety and effectiveness, and associated with better adherence to medical advice and better health outcomes [9]. However, data on the relationship between patient experience and staffing levels is conflicting, with some studies showing a positive association between patient satisfaction and nursing levels [10–12] and others not [13, 14]. These differences may stem from limitations of extant studies, including a tendency to measure staffing levels at hospital, rather than ward level, and a failure to control for confounding factors such as the influence of other health professionals concurrently involved in patient care [15].

The majority of research on the effect of staffing levels has been conducted on adults of all ages, rather than older adults, despite the fact that in 2014–2015 62% of hospital bed days were occupied by patients over 65 years old [16], and older adults have specialised needs, including basic nursing care and support for cognitive impairment, which are not always met [17]. A systematic review of older adults’ experiences of hospital care [18] reported that they tended to take technical aspects of care for granted and were more concerned about whether staff have time to provide personal care. This concept of staff’s ‘time to care’ was highlighted in the Royal College of Nursing report on older adult care [5], which noted the importance for nursing staff of having time to address the special care needs of elderly patients and provide compassionate care. This suggests that perhaps nursing staff’s ‘time to care’ for patients may be as important as staffing levels in enhancing patient experience of the care they receive.

The aim of the present study was to evaluate the association between staffing levels, healthcare assistant (HCA) to registered nursing staff ratios, staff’s perceived ‘time to care’ and older adult patient satisfaction with hospital care. The research questions were:

1. What is the association between older adult patient satisfaction and:
  the number of patients per staff of any type.
  the number of patients per registered nurse or HCA.
  the number of HCAs per registered nurse?
2. Is any association between staffing ratios and older adult patient satisfaction mediated by staff’s perceived time to care for patients?

## Methods

### Design

This study was a cross-sectional analysis of ward and individual-level predictors of older adult patient satisfaction, using data from 123 acute and community wards in England, United Kingdom (UK) collected by the Quality Mark for Elder-Friendly Hospital Wards programme at the UK Royal College of Psychiatrists between September 2012 and December 2016. This is a subscription-based quality improvement programme aiming to help acute and community hospitals to improve the quality of care they provide for people over the age of 65 [19]. Only data from the start of wards’ participation in the Quality Mark – i.e. before they had attempted to make improvements to patient care through participating in the programme – was used.

### Setting and eligibility criteria

All wards in all acute and community medical hospitals in England were invited to take part in the Quality Mark for Elder-Friendly Hospital Wards programme between 2012 and 2016. The study included any patients over the age of 65 whom staff had deemed medically fit for discharge after a stay of at least two nights, and any staff directly involved in the care of patients aged over 65 in hospital, including HCAs, registered nurses, medical staff and members of the multidisciplinary team. Patients who were unwilling to complete the questionnaire or who did not have the mental capacity to understand the purpose of the questionnaire or to complete the questionnaire (with or without help from friends or family) were excluded.

### Recruitment and data collection

The management staff of wards which agreed to take part were sent detailed information about the programme, and were asked to nominate a member of staff on each shift to be the patient questionnaire lead, who would be responsible for identifying eligible patients and handing out questionnaires. Leaflets about the programme were also handed out to patients and posters were displayed around the ward. Patients were informed that the questionnaire would be anonymous and that their responses would not affect their future care. Ward staff were asked not to help patients complete questionnaires, however, friends, family, volunteers or patient advocates were able to help when needed. Patients were also given the option of completing questionnaires at home following discharge and returning them directly to the Royal College of Psychiatrists.

Wards were given a target to obtain questionnaires from at least 40% of eligible patients during a three-month data collection period. Ward managers were also asked to encourage all eligible staff on their ward to complete the Staff Feedback questionnaire via the Royal College of Psychiatrists’ website during this period. Staff were informed that their responses would be anonymised. Wards staff were asked to aim to get a response from at least 50% of eligible staff. Finally, within the 3-month data collection period, ward managers were asked to complete a record of actual staffing levels over a 4-week period. Ward managers could submit staffing data either in paper format by post, or electronically via email or fax.

To maximise generalisability of findings, we aimed to include all eligible consenting participants across England, and hence sample size was determined by numbers of wards taking part in the Quality Mark and numbers of consenting patients within each ward, rather than by any pre-specified target sample size.

### Measures

#### Patient feedback questionnaire

The Patient Feedback Questionnaire is a 22-item self-report questionnaire developed by the Royal College of Psychiatrists [19]. Patients are asked to rate their satisfaction with: comfort on the ward (5 items), eating and drinking (5 items), support from staff (3 items), getting help when I need it (5 items), privacy and dignity (4 items); using a Likert scale for each item ranging from 0 (Strongly disagree) to 4 (Strongly agree). A total score is then calculated to give an index of each patient’s overall satisfaction with care on the ward. The internal consistency of the 22 items in the current study was high (Cronbach’s alpha = 0.92).

#### Staff feedback questionnaire

The Staff Feedback Questionnaire is a 38-item self-report questionnaire developed by the Royal College of Psychiatrists [19]. Staff are asked to rate the extent to which they feel morale, leadership and teamwork is encouraged on the ward (7 items), and the extent to which they feel they have ‘time to care’ i.e. provide patients and their families with emotional and practical support and information (5 items), are given the skills to care i.e. the training and supervision to understand and care for common health difficulties amongst older adults and to communicate about these with patients and involve them in decisions about their care (8 items), have access to support from the wider hospital e.g. interpreting and advocacy services (5 items), and have access to relevant training (12 items). For the present study, the mean of each staff member’s ratings of 4 of the 5 ‘time to care’ items (excluding 1 item relating to time to discuss and explain care and treatment to patients’ families, as not all participating patients had family involved in their care) was calculated in order to generate an index of each staff member’s perceived time to care for patients. The 4 items used were: I have enough time to provide practical assistance to patients when they need it, e.g. support walking to the toilet, At mealtimes, I have enough time to ensure patients who need assistance receive it, I have enough time to provide patients with reassurance when they need it, I have enough time to discuss and explain care and treatment to patients. Staff rated these items using a Likert scale ranging from 0 (Strongly disagree) to 4 (Strongly agree). The internal consistency of the 4 items used in the current study was high (Cronbach’s alpha = 0.88).

#### Staffing levels

For each ward, information provided by ward managers on staff and patient numbers over a 4 week period was used to calculate: the mean overall patient to staff ratio, whereby a larger ratio indicated more patients per staff member; patient to registered nurse ratio, whereby a larger ratio indicated more patients per each registered nurse; and HCA to registered nursing staff ratio, whereby a larger ratio indicated more HCAs per registered nurse.

#### Ward type

Ward managers provided information on the types of patients and conditions treated on their ward, which were classified into older-adult (age 65 and older) versus all ages, and acute versus post-acute (transitional, intermediate, rehabilitation, discharge) or community wards.

### Data analysis

All analysis was conducted using STATA/ SE version 14.2 [20]. Predictors of patient satisfaction were modelled using linear regression, with robust standard errors to account for the non-normal distribution of patient satisfaction. Predictors included patient sex, and the interaction effect of patient sex and staffing levels as potential confounders, as these have been found to predict satisfaction amongst older adults in previous research [21]. Patients with missing data on a predictor of interest were omitted from the analysis of the effect of that predictor; a pro-rated total satisfaction score was used for patients with < 10% of data missing on the dependent variable. As an initial multi-level mixed effects regression model indicated significant clustering of satisfaction ratings within wards, all subsequent analyses therefore used multilevel modelling with a random effect for ward, in order to adjust the models for differences between wards in patient satisfaction. Multiple regression models were used to evaluate the effect of staffing on patient satisfaction, adjusting for any patient and ward characteristics found to predict satisfaction at *p* < 0.05 in simple regression models. Multilevel multiple mediation models based on Krull and McKinnon’s product of coefficients method were used to evaluate whether any association between staffing ratios and older adult patient satisfaction was mediated by staff’s perceived time to care for patients [22, 23] using bootstrapping with 500 replications to obtain bias-corrected confidence intervals.

## Results

### Description of the sample

The final sample consisted of data from 4928 patients and 2237 staff from 123 hospital wards. The majority of participating wards were acute general medical wards (83%), whilst 10% provided post-acute care (rehabilitation/ transitional/intermediate/pre-discharge care) and 7% provided community care. Roughly equal numbers provided care only for older adults (49%) or for mixed ages (51%).There were more males than females in the patient sample (55% male, 35% female, 10% missing data), and a large majority of the patient sample described themselves as white British (84% white British, 4% white other, 5% black or minority ethnic, 7% missing data). Patient ages ranged from 65 years old to above 95 years old, with 21% aged 65 to 74, 38% aged 75 to 84, 31% aged 85 to 94, and 4% aged 95 years or older (6% missing data). The staff sample consisted of 88 medical staff, 1145 qualified nurses and 1004 unqualified nursing staff. All 123 wards met their target for data collection (i.e. at least 40% of eligible patients during the data collection period, including a minimum of 25 patients, and at least 50% of eligible staff), with the exception of 4 wards which provided a below-target number of staff questionnaires, and 12 wards which provided a below-target number of patient questionnaires.

### Patient satisfaction, staff time to care and staffing ratios

The mean patient-rated satisfaction with their care on the Patient Feedback Questionnaire -was 65.5 (SD = 11.08, *N* = 4134), with scores ranging from 3 to 84, out of a possible maximum of 88. The mean patient: staff ratio on participating wards was 3.73:1 (SD = 0.66, *N* = 115 wards). The mean patient: registered nurse ratio was 7.95: 1 (SD = 2.36, *N* = 115 wards). The mean patient: HCA ratio was 7.86:1 (SD = 2.80, *N* = 115 wards). The mean HCA: registered nurse ratio was 1.11: 1 (SD = 0.55, *N* = 115 wards). The mean staff-rated time to care on the Staff Feedback Questionnaire - was 2.54 (SD = 0.91, *N* = 2237 staff), with scores ranging from 0 to 4, out of a possible maximum of 4. Mean perceived time to care was significantly lower for HCAs than for medical staff (β = − 0.23, 95% CI − 0.41 to - 0.05, *p* = 0.01), and significantly lower for registered nurses than for HCAs (β = − 0.27, 95% CI − 0.34 to − 0.19, *p* < 0.001).

### Patient and ward-level predictors of patient satisfaction

A multi-level mixed effects regression model indicated significant clustering of satisfaction ratings within wards (likelihood ratio test Χ2 = 378.8, *p* < 0.01), with differences between wards explaining 14% of the variance between patients in satisfaction ratings. All subsequent analyses therefore included a random effect for ward identity.

Unadjusted predictors of patient satisfaction are shown in Table 1. Patient satisfaction ratings did not differ by sex nor by age or ethnicity of the respondent, and nor was there any interaction effect of patient sex and staffing ratios. Nor did ratings differ between older-age and mixed-age, or between acute and post-acute wards. However, satisfaction was significantly lower on acute than on community wards (β = − 4.84, 95% CI − 9.25 to − 1.42, *p* < 0.01). Analyses of the effect of staffing ratios and perceived time to care were therefore adjusted for ward type (acute vs. post-acute vs. community).

**Table 1:** Predictors of patient satisfaction (unadjusted)

Predictor	N wards	N patients	β^**a**^	95% CI	p
**Patient characteristics**					
Patient sex					
Male (vs. female)	122	3735	0.26	−0.49 to 1.01	0.50
Patient age (years)	122	3933	−0.17	−0.62 to 0.27	0.45
Patient ethnicity					
Black & ethnic minority (vs. white)	122	3879	−0.41	−1.88 to 1.07	0.59
**Ward characteristics**					
Ward age range					
Mixed-age (vs. older-adult)	122	4134	−1.06	−2.68 to 0.56	0.20
Ward type					
Post-acute (vs. acute)	122	4134	1.37	−1.37 to 4.12	0.33
Community (vs. acute)			4.84	1.42 to 8.25	< 0.01
Number of patients per staff member	115	4044	−1.43	−2.74 to −0.14	0.03
Number of patients per registered nurse	115	4044	0.16	−0.22 to 0.54	0.40
Number of patients per HCA	115	4044	−0.42	−0.66 to − 0.17	< 0.01
Number of HCAs per registered nurse	115	4044	1.91	0.26 to 3.56	0.02
Interaction between number of patients per registered nurse and number of patients per HCA	115	4044	−0.09	−0.24 to 0.07	0.26
Interaction between patient sex and number of patients per staff member	115	3653	−0.31	−1.44 to 0.82	0.59
Interaction between patient sex and number of patients per nurse	115	3653	0.05	−0.26 to 0.37	0.75
Interaction between patient sex and number of patients per HCA	115	3653	−0.13	−0.40 to 0.14	0.35
Mean staff perceived time to care	117	4051	4.42	2.26 to 6.58	< 0.01

^a^ All analyses were multi-level with a random effect for Ward identity to account for clustering of patient satisfaction ratings within wards

### The association between staffing ratios and patient satisfaction

Whilst in unadjusted models (Table 1), higher overall patient to staff ratios and lower HCA to registered nurse ratios predicted poorer patient satisfaction, these effects were no longer significant after adjusting for ward type (Table 2), suggesting confounding by higher levels of patient satisfaction in community wards. By contrast, both in unadjusted models (Table 1), and in models adjusting for ward type (Table 2), a higher number of patients per HCA was associated with poorer patient satisfaction (adjusted β = − 0.32, 95% CI − 0.55 to 0.10, *p* < 0.01). In both unadjusted and adjusted models, the number of patients per registered nurse was not associated with patient satisfaction, and the interaction between patients per registered nurse and patients per HCA was non-significant.

**Table 2:** Staffing and time to care as predictors of patient satisfaction (adjusted for ward type)

Predictor	N wards	N patients	β^a,b^	95% CI	p
Number of patients per staff member	115	4044	−1.07	−2.36 to 0.17	0.10
Number of patients per registered nurse	115	4044	0.11	−0.25 to 0.47	0.54
Number of patients per HCA	115	4044	−0.32	−0.55 to − 0.10	< 0.01
Number of HCAs per registered nurse	115	4044	1.40	−0.19 to 2.99	0.09
Interaction between number of patients per registered nurse and number of patients per HCA	115	4044	−0.07	−0.22 to 0.07	0.33
Mean staff perceived time to care	117	4051	4.23	2.09 to 6.37	< 0.01

^a^ All analyses were multi-level with a random effect for Ward identity to account for clustering of patient satisfaction ratings within wards

^b^ All analyses were adjusted for ward type: acute vs. post-acute vs. community

### Mediation by staff perceived time to care

In both adjusted and unadjusted models, patients reported greater satisfaction with their care if they were treated in wards in which staff reported having more time to care for patients (Tables 1 and 2). Adjusting for ward and ward type, there was a significant indirect negative effect of the number of patients per HCA on patient satisfaction, via mean staff perceived time to care for patients (adjusted β = − 0.10, bias-corrected 95% CI − 1.16 to − 0.02). Mean staff perceived time to care was found to mediate 32% of the effect of patient: HCA ratios on patient satisfaction.

## Discussion

### Summary of Main findings

To our knowledge this is the first study to look at the association between older adult patient satisfaction, nursing staffing levels and staff’s perceived time to care on hospital and community wards in the UK. We found that older adult patient satisfaction did not differ by sex, age or ethnicity, but was higher in community wards, in wards with more HCAs per patient, and in wards where staff reported having more time to care for patients. In contrast to previous research in older adults [21], we did not find greater satisfaction in female patients, nor any interaction effect of patient sex and staffing ratios. About a third of the effect of patient: HCA ratios on satisfaction was mediated by higher overall staff perceived time to care for patients. By contrast, the number of patients per registered nurse was not associated with patient satisfaction.

### Strengths and limitations

Strengths of the study include the large multi-site nationally representative sample and the use of ward-level data, enabling a more sensitive representation of actual staffing numbers than in previous research which has relied on hospital-level data [15]. Additionally, the specific focus of our study on older adults allowed important differences specific to this population to be elucidated. Additionally, we have adjusted for differences in patient satisfaction between wards and ward type, and have evaluated a potential mediator of the effect of staffing ratios on satisfaction, which previous studies have not done.

Limitations include the use of cross-sectional data, preventing causal inference, and the findings may not be generalisable beyond the UK healthcare system. Additionally, whilst attempts were made to control for confounding factors, there may be other confounding variables that were not measured or controlled for. Furthermore, the use of data from the Royal College of Psychiatrists Quality Mark programme may have affected the representativeness of the sample as it may be that wards applying for the programme are more likely than non-applicants to be well-led, high-achieving wards, who have self-selected because they think they are already ‘elder-friendly’. Relatedly, staff may have given more positive responses about having time to care if they believed their responses would affect whether or not they achieved the Quality Mark; we aimed to circumvent this by using data only from the baseline round of the programme, which did not influence Quality Mark status. Patient sex was unbalanced in the sample, with more males than females responding. However, we found no effect of patient sex nor interaction effect of patient sex and staffing ratios on satisfaction, suggesting the potential sex imbalance did not overly influence our findings.

### Interpretation of findings in the context of previous research

There was a large variation in patient satisfaction and staffing ratios between wards, with many wards falling below the Royal College of Nursing’s recommendation of at least one registered nurse for seven patients, and below their recommendation of at least one registered nurse for every HCA. This variation in staffing was also found by Aiken et al. [24], who noted that nurses in some hospitals were caring for twice as many patients as nurses in other hospitals. Our findings correspond to previous findings amongst mixed-age adult acute hospital inpatients, whereby both higher staff: patient ratios and greater perceived time to care for patients were associated with higher patient satisfaction [10–12, 24–27]. A novel finding from our data not shown in previous research was that, after taking account of differences in patient satisfaction between wards and ward types, only greater availability of HCAs predicted patient satisfaction, whilst the availability of registered nurses did not affect patient satisfaction, and that the effect of HCA availability was mediated by all staff types feeling that they had more time available to care for patients. This is contrary to findings on the importance of registered nursing staff availability for satisfaction amongst mixed-age patients [14, 28, 29] and also stands in direct contrast to another recent UK study amongst older adult hospital patients, which found that higher HCA staffing levels in the context of low registered nurse levels were associated with poorer quality staff-patient interactions [30]. However, our finding is broadly in keeping with a previous study conducted among older adults showing that a lower number of HCAs relative to registered nurses was associated with poorer satisfaction [27].

A possible explanation for the importance of HCAs in our findings may lie in the suggestion that older adults prioritise different aspects of care than younger adults. In practice, HCAs provide the majority of direct bedside care [31, 32], focussing on attending to patients’ physical comfort, helping them to eat and move about, and talking and listening to them [32].Older adults have complex needs, often needing help with activities of daily living, and more time is needed for communication due to sensory and cognitive impairments [5, 33]. Therefore, older patients may value HCAs for their ability to help more with personal and relational areas of care. The vital importance of addressing deficiencies in this area has been emphasised by reports on failings in patient care in the UK [1–4]. Additionally, our mediation analysis suggests that when there are more healthcare assistants available on a ward, this has a knock-on positive impact on other type of staff too, whereby all staff feel that they have more time communicate with and offer practical support to patients, leading to higher patient satisfaction. This finding is in direct contrast to previous suggestions that high HCA levels lead to poorer quality interactions with patients due to lack of supervision by registered nurses [30]. The importance of all staff types being able to communicate positively with patients is highlighted in the most recent UK National Clinical Audit of Dementia Care, which found that a whole ward team approach is needed in order to be able to truly deliver patient-centred care [4].

### Implications for health services and further research

The study findings in general reinforce the importance of adequate staff to patient ratios for patient satisfaction and for the provision of good quality, safe care on older adult wards [5, 6], but suggest that the role of HCAs is of particular importance for promoting a positive experience of care amongst older adults. Currently, national guidance on staffing does not specify a minimum HCA to patient ratio [5, 6]. However, caution is merited since it has been shown that adequate registered nursing staffing is vital for reducing patient mortality whereas HCA staffing has less effect on this crucial outcome [14, 34]; whilst HCAs may be important for patient satisfaction, they cannot be safely substituted for registered nurses, and it is therefore important that national guidelines continue to emphasise standards for minimum numbers of registered nurses per patient and per HCA.

Future research should seek to identify the ideal ratio of HCAs to patients and registered nurses in order to optimise a range of outcomes amongst older adult patients, prioritising key outcomes such as mortality but not neglecting to consider the potentially positive effects of HCA staffing on patient experience. Further research is also needed to better understand how and why HCAs affect patient experience, and how their contribution to the ward affects other staff’s ability to engage in patient-centred compassionate care. In the present study, staff’s perceived time to care only partially mediated the effect of HCA staffing ratios on patient satisfaction. It is possible that patients’ perceptions of staff-patient interactions could be a more important mediator of satisfaction than staff’s perceptions; it would be valuable to know what patients value about their communications with HCAs and how this differs from communications with other types of staff. Additionally, other factors such as the value to older adult patients of the specific types of tasks carried out by HCAs (such as assistance with eating, dressing, washing and toileting) may be important. Furthermore, the Quality Mark did not collect data on medical staffing ratios; future research should investigate the effect of medical staffing and how this interacts with nursing and HCA staff availability and perceived time to care. Additionally, registered nurses reported having less time to communicate and assist with basic care of patients than HCAs, despite arguably being better qualified to explain patients’ condition and treatment to them than HCAs. Further research should investigate what factors influence staff’s ability to spend time communicating with patients, and why this is more difficult for registered nurses than HCAs. Such research should also identify how ward leadership and priorities, and staff training and support structures, can facilitate staff to spend more time communicating with patients, and whether nursing staff can be freed from non-essential duties such as administration in order to spend more time applying their knowledge and communication skills in this vital capacity.

## Conclusions

Using survey data from a large and nationally representative sample of medical wards across the United Kingdom, we have shown that availability of HCAs– but not availability of registered nurses – is associated with higher patient satisfaction amongst older adults, and that this may partly be due to higher availability of healthcare assistants contributing to all ward staff feeling more able to spend time with patients. Older adult hospital patients may particularly value the type of care provided by HCAs, such as basic personal care and supportive communication. National staffing guidelines currently do not specify a minimum HCA to patient ratio, and our findings suggest that specifying such standards for HCA staffing could be important for patient satisfaction. However, high availability of registered nurses has been shown in other research to be vital for ensuring quality and safety of patient care and it is therefore important to continue to emphasise the importance of minimum registered nurse to patient and registered nurse to HCA ratios. Future research should seek to identify the ideal balance of registered nurses and healthcare assistants for optimising a range of outcomes amongst older adult patients.

## Data Availability

The datasets used and/or analysed during the current study are available from the corresponding author on reasonable request.

## Abbreviations

HCA: Healthcare assistant
UK: United Kingdom
